# Editorial: The Emerging Role of Interdisciplinarity in Clinical Psychoanalysis

**DOI:** 10.3389/fpsyg.2021.809546

**Published:** 2021-12-09

**Authors:** Aner Govrin, Jon Mills, Ronald C. Naso

**Affiliations:** ^1^The Program for Hermenutics and Cultural Studies, Bar-Ilan University, Ramat Gan, Israel; ^2^Gordon F. Derner School of Psychology, Adelphi University, New York, NY, United States

**Keywords:** psychoanalysis, infant research, neuropsychoanalysis, CBT, attachment theory

Psychoanalysis is not isolated from the scientific and cultural world. All are intertwined, nourishing and shaping each other and having a dialogue of mutual criticism. Related disciplines such as infant observation, neuroscience, attachment theory, psychotherapy research, emotional regulation research, even philosophy have penetrated psychoanalytic thought in myriad ways. Findings from other disciplines have improved psychoanalytic knowledge on the structure of the mind, the transference, the role of dreams and affect in therapy, memory processes, trauma, and many other subjects.

This special issue provides a collection of 11 articles that deepen and develop our understanding of the ways and means that clinical psychoanalysis intersects with other disciplines. The collection draws on the work of 12 researchers from different countries worldwide. Each article illustrates various connections between psychoanalysis and other fields of knowledge, including infant development, neuroscience, and philosophy.

Its purpose is to bring readers the rich and varied writing of psychodynamic psychotherapists who crossed the border to bring informative knowledge from other fields. We hope to encourage and stimulate a research discourse that focuses on interdisciplinary psychoanalytic thinking and fruitful dialogue between psychoanalysis and other disciplines.

In “Interoception Disorder and Insular Cortex Abnormalities in Schizophrenia: A New Perspective Between Psychoanalysis and Neuroscience,” Tran The et al. indicate new directions for research in the study of the correlations between the functional abnormalities of the insular cortex and the positive symptomatology of schizophrenia, with regard to the Freudian hypothesis of the primary character of psychopathology linked with interoceptive perception in relation to delusional ideas and auditory hallucinations.

In “The Emerging Role of Interdisciplinarity in Clinical Psychoanalysis,” Steinmair and Löffler-Stastka describe several attempts that have been made to operationalize psychoanalytic core competencies and a transparent analysis of the psychoanalytic mindset. These attempts include neuroimaging studies, default mode network (DMN), autobiographical memory, emotion processing, Facial Action Coding System (FACS), and affective linkage of long-term memory. They show how integrating different viewpoints and analysis with various methods lead to a more complete image acquired by interdisciplinary communication and collaboration rather than fragmentation of resources.

In “Narcissistic Personality Disorder: Are Psychodynamic Theories and the Alternative DSM-5 Model for Personality Disorders Finally Going to Meet?” Schalkwijk et al. integrate contemporary psychodynamic concepts of narcissism, and the diagnostic concept of narcissism in the Model for Personality Disorders (AMPD). They argue that the combined dimensional and trait conceptualization of AMPD opens the door to new integrated diagnostic perspectives, including both internal and interpersonal functioning.

In “Dreams and Trauma Changes in the Manifest Dreams in Psychoanalytic Treatments—A Psychoanalytic Outcome Measure,” Fischmann et al. use the model of dream generation by Moser and von Zeppelin, which has integrated a large interdisciplinary knowledge base of contemporary dream and sleep research with psychoanalytic accounts of dreaming.

In “Psychoanalysis and Interdisciplinarity With Non-analytic Psychotherapeutic Approaches Through the Lens of Dialectics,” Herzovich and Govrin refer to Hegelian dialectics in an attempt to offer an alternative approach to interdisciplinarity in clinical psychoanalysis.

In “The Emergence of Psychoanalytic Metaneuropsychology: A Neuropsychoanalytically Informed Reconsideration of Early Psychic Development,” Mellor uses infant research and neuroscience to shed new light on some of the major disagreements that separated the Viennese and the London Kleinians during the British Psychoanalytical Society's Controversial Discussions.

In “The Historical Influence of Psychoanalytic Concepts in the Understanding of Brain Injury Survivors as Psychological Patients,” Salas reviews key psychoanalytic ideas that have influenced the understanding of brain injury as a psychological problem, and of brain injury survivors as patients with unique psychological rehabilitation needs.

In “Project for a Spatiotemporal Neuroscience”—Brain and Psyche Share Their Topography and Dynamic,” Northoff and Scalabrini posit that Spatiotemporal Neuroscience, which focuses on the spatial topography and temporal dynamic of the brain's neural activity, provides a model of the brain that is more or less analogous to Freud's view of the psyche. They show how practically, the “Project for a Spatiotemporal Neuroscience” lays the groundwork for a novel form of neuroscientific informed psychotherapy, namely Spatiotemporal Psychotherapy.

In “The Changeable Positioning of the Couch and Repositioning to Face-to-Face Arrangement to Facilitate the Experience of Being,” Ünsalver et al. focus on eye gaze and the experience of being seen to support the importance of eye gaze in the psychoanalytic encounter. The authors argue that sometimes it seems necessary for the analysand to re-establish eye contact with the new caregiver to experience attunement to reconstruct and repair early object relations.

In “Clinical Applications of Neuropsychoanalysis: Hypotheses Toward an Integrative Model,” Mosri explores clinical applications of neuropsychoanalysis mainly based on affective neuroscience to propose an analysis of emotions that may contribute to the gradual development of a neuropsychoanalytically informed psychotherapy.

In “The Clinical Relevance of Interdisciplinary Research on Affect Regulation in the Analytic Relationship,” Altimir and Jiménez study the simultaneous capture and analysis of the verbal contents and the interaction of gestures and glances as an expression of the implicit unconscious exchange.

In “Reformulated Object Relations Theory: A Bridge Between Clinical Psychoanalysis, Psychotherapy Integration, and the Understanding and Treatment of Suicidal Depression” Shahar aims to show how a reformulation of object relations theory (RORT) using (neuro-)psychological science may enhance a clinical-psychoanalytic understanding and treatment of suicidal depression, which constitutes one of the most formidable health challenges of our time.

## Author Contributions

AG was the central author. RN and JM contributed in reading, editing, and adding to the text. All authors contributed to the article and approved the submitted version.

## Conflict of Interest

The authors declare that the research was conducted in the absence of any commercial or financial relationships that could be construed as a potential conflict of interest.

## Publisher's Note

All claims expressed in this article are solely those of the authors and do not necessarily represent those of their affiliated organizations, or those of the publisher, the editors and the reviewers. Any product that may be evaluated in this article, or claim that may be made by its manufacturer, is not guaranteed or endorsed by the publisher.

